# Feto-placental blood vessel development

**DOI:** 10.1242/dev.204838

**Published:** 2025-06-02

**Authors:** Jacinta I. Kalisch-Smith

**Affiliations:** Institute for Developmental and Regenerative Medicine, Department of Physiology, Anatomy and Genetics, The University of Oxford, Old Road Campus, Headington, Oxford OX3 7TY, UK

**Keywords:** Allantois, Feto–placental blood vessels, Endothelial cells, Pregnancy complications, Congenital heart defects

## Abstract

Development of the feto–placental blood vessels (human), or chorio–allantoic vasculature (mouse), is crucial for embryonic and fetal survival. While the processes governing embryonic vascular development are fairly well established, our understanding of feto–placental vascular formation is lagging decades behind. There are many unanswered questions in the field regarding potential progenitor populations, the timing of arterio–venous differentiation, the molecular cues that induce angiogenesis and the sources of these factors. In humans, particularly, there is little information on first-trimester placental vascular development or what pathologies may be caused by poor vascularisation. This Review discusses known processes of feto–placental blood vessel development in mice and humans, including their progenitors and derivatives (with their molecular markers), genetic knockouts and associated vascular phenotypes, trophoblast-endothelial signalling, co-occurrence with embryonic heart defects, genetic tools and imaging modalities targeting these vessels and pathologies that are impacted by vascular defects. Recent insight into early human placental vascularisation suggests it is more similar to the mouse than previously appreciated.

## Teaser

**Summary:** This Review contains an overview of placental vascular development and pathologies in mice and humans and highlights key remaining questions in the field.

## Introduction

Formation of the placental circulation is crucial for fetal survival and a successful pregnancy. In both humans and mice, the placental circulation is made up of two separate blood supplies, one maternal and one fetal. Maternal nutrients and gases must cross the placental barrier to be delivered to the fetal circulation. While the maternal side has large spiral arteries carrying maternal blood to the sinusoids of the placenta, on the fetal side, the feto–placental blood vessels transport nutrients and gases from the maternal circulation to the fetus. Despite the important role these placental vessels play during development, our understanding of their developmental origin, including the progenitor cells that give rise to them and the genetic pathways that control their formation, is decades behind that of the embryonic vasculature in both mice and humans. Poor adoption of placental vasculature research by embryonic vascular biologists and clinicians is likely due to several reasons. Firstly, the placental circulation is counter-intuitive, in that the placental arteries carry deoxygenated blood whereas the embryonic arteries carry oxygenated blood. Secondly, until recently, we have lacked genetic markers and genetic tools to compare placental endothelial cells (ECs) to embryonic ECs in mouse models. Finally, in humans, we are similarly limited by access to early post-implantation stage tissue, technology and resolution of imaging modalities to track their formation during pregnancy. Given these caveats in diagnostic imaging, the prediction and treatment of vascular-associated disorders are lacking. Furthermore, formation of these placental vessels in the mouse occurs in a series of stages for embryonic viability; however, such checkpoints in human development are yet to be investigated.

**Fig. 1.**
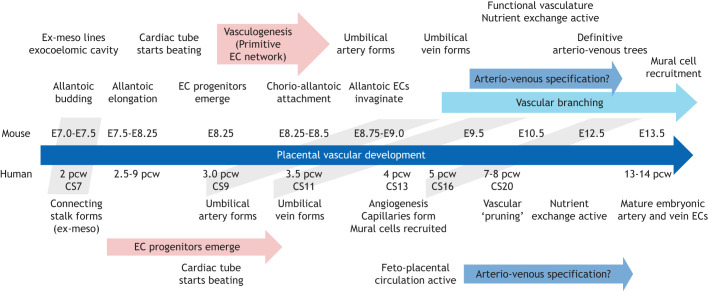
**Timeline of mouse versus human placental vascular development.** Grey boxes indicate equivalent stages in mouse versus human development. CS, Carnegie stage; E, embryonic day; EC, endothelial cell; Ex-meso, extra-embryonic mesoderm; pcw, post-conception weeks.

The placenta contains cells from three different cell lineages: maternal uterine cells making up the decidua (uterine lining), trophoblast subtypes derived from the trophectoderm (outermost cellular layer of the early embryo) and extra-embryonic mesoderm (originating from the epiblast; see Glossary, Box 1), which contributes to the feto–placental vasculature (see Glossary, Box 1). The vessels of the placenta are located within structures known as the ‘labyrinth’ in the mouse and ‘villi’ in humans (see Glossary, Box 1). Materno–fetal exchange occurs by embryonic day (E)10.5 in mice and by ∼10 post-conception weeks (pcw; see Glossary, Box 1) in humans, based on experiments examining maternal spiral arterial blood flow into the intervillous space and increased oxygen tension (Jauniaux et al., 2000) (Fig. 1).

**Box 1. Glossary**

**Allantois (mouse).** Extrusion of extra-embryonic mesoderm that extends towards the chorion. Origin of the umbilical and chorio–allantoic (labyrinth) vasculature.

**Allantois (human).** The ‘allantois’ or ‘allantoic diverticulum’ in humans is a separate structure from the umbilical cord vessels and ‘feto–placental’ vessels that derive from the connecting stalk. The connecting stalk is equivalent to the mouse allantois. The human allantois forms a singular extension from the hindgut, which exists temporarily between the two umbilical arteries. It regresses from the umbilical cord to connect the hindgut to the cloaca and eventually becomes the ‘urachus’ or ‘umbilical ligament’.

**Angioblast.** Derived from mesoderm, this progenitor gives rise to both endothelium and hematopoietic cells.

**Chorio–allantoic vasculature (mouse).** Allantois-derived placental vessels that connect to the umbilical cord, which form the ‘fetal blood spaces’. Equivalent to the feto–placental blood vessels in humans.

**Chorio–vitelline vasculature.** Yolk sac blood vessels, including the vitelline artery and vein.

**Chorion (mouse).** Progenitor population to syncytiotrophoblast layer cells.

**Chorionic plate.** Region where the umbilical cord vessels enter the placenta, and branch to create the villous trees (arterial and venous).

**Connecting stalk (human).** Extra-embryonic protrusion (likely extra-embryonic mesoderm), which contributes to the umbilical and feto–placental vasculature in humans. Equivalent to the mouse allantois.

**Feto–placental vasculature (human).** Placental blood vessels that connect to umbilical cord vessels.

**Labyrinth (mouse).** Major zone of mouse placenta containing chorio–allantoic blood vessels (endothelial cells), as well as allantois derivatives (e.g. pericytes, smooth muscle cells, stromal cells) and trophoblast cell types (e.g. syncytiotrophoblast layers I and II, sinusoidal trophoblast giant cells) making up the placental barrier.

**Mesenchyme.** In the placenta, ‘mesenchyme’ has been used in various settings to describe the cellular morphology of connective tissue. In humans, it has been used to describe loosely organised villous stromal cells (a range of cell types of unknown origin), while in the mouse it has been used for poorly described allantoic mesoderm derivatives (e.g. stromal cells), as well as a thin mesothelial layer (an extra-embryonic mesoderm-derived tissue that lines the exocoelomic cavity and attaches to the chorion), termed ‘chorionic mesenchyme’ or ‘chorionic mesothelium’.

**Mesoderm.** Germ layer that gives rise to the embryonic vasculature, blood, bone, connective tissue and muscle lineages, amongst others, including in the extra-embryonic mesoderm (giving rise to allantoic mesoderm in mice).

**Post-conception weeks (human).** Describes time in human gestation post-conception, not to be confused with ‘gestation weeks’, which is the time since the last menstrual period and includes an additional 2 weeks before conception.

**Stroma (human).** Used to describe mesenchymal cells in the human villi, making up the ‘stromal core’ and consisting of hematopoietic cells (i.e. macrophages/Hofbauer cells), fibroblasts and pericytes.

**Umbilical cord.** Extra-embryonic derived structure that contains the umbilical vein and artery (mouse)/arteries (human).

**Villi (human).** Location of feto-placental blood vessels, which also contains villous ‘stroma’ and trophoblast cells in finger-like projections extending from the chorionic plate.

**Fig. 2.**
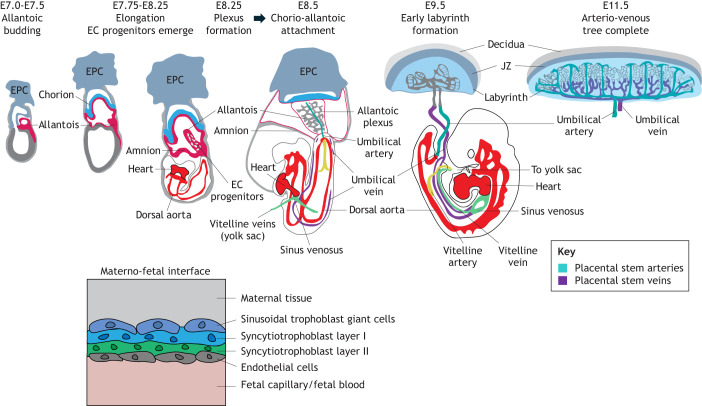
**Mouse labyrinthine feto–placental blood vessel development.** The allantois (magenta) buds from extra-embryonic mesoderm by E7.5 and extends towards the chorion (blue) while differentiating into endothelial (EC) progenitors. Continued extension of the allantois creates a primary endothelial plexus by E8.25. A centrally located umbilical artery (teal) can be seen by E8.5, the time point of chorio–allantoic attachment. Following this, allantois-derived ECs bud and branch to create the labyrinthine vasculature (grey). The umbilical vein (purple) is present in the embryo by E8.5 but can only be viewed in the allantois/umbilical cord by E9.5, when the early labyrinth vasculature is already apparent. By E11.5, an immature arterio–venous tree can be observed. Cells making up the placental barrier can be seen (bottom left). Diagrams based on images from Arora et al. (2012), Simmons (2013), Walls et al. (2008), Thomas et al. (2021) and Kalisch-Smith et al. (2022). Decidua (light grey), EPC (grey blue), sinus venosus (green), placental veins (purple), placental arteries (teal), dorsal aorta and embryonic circulation (red), vitelline vein (green), vitelline artery (yellow). EPC, ecto-placental cone; JZ, junctional zone.

**Fig. 3.**
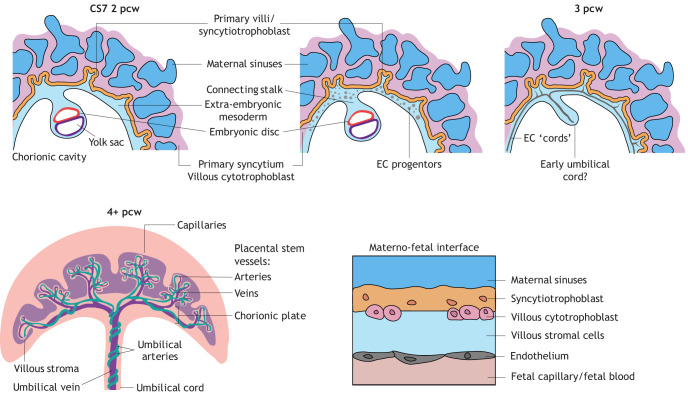
**Human villous feto–placental blood vessel development.** Connecting stalk extra-embryonic mesoderm (light blue), which is the precursor to feto–placental blood vessels and umbilical cord, invades into the primary syncytium containing villous cytotrophoblast (magenta) from 2 post-conception weeks (pcw). From 3 pcw, endothelial cell (EC) progenitors (grey circles) and EC ‘cords’ (grey lines) can be found, which generate the feto–placental vasculature. Capillaries are located in the peripheral part of the primary villi (orange), while stem vessels connect to large chorionic plate vessels that attach to the umbilical cord. Cells making up the placental barrier can be seen (bottom right). Figures based on Ross and Boroviak (2020), Knöfler et al. (2019) and Thomas et al. (2021).

In the mouse, the allantois (see Glossary, Box 1) gives rise to the umbilical artery and vein, in addition to the placental vessels that connect to them, to create the ‘chorio–allantoic’ vasculature (see Glossary Box 1) (Fig. 2). Before this vasculature becomes functional at E10.5, the ‘chorio–vitelline’ vasculature of the yolk sac provides initial nutrients and gas exchange. The yolk-sac vasculature envelops the embryo in the mouse, whereas in the human it is a sac adjacent to the connecting stalk (see Glossary, Box 1). In humans, the allantois (or ‘allantoic diverticulum’; see Glossary, Box 1) is a separate structure from the umbilical cord (see Glossary, Box 1) vessels and the ‘feto–placental’ vessels, which both derive from the connecting stalk (Fig. 3). Here, the human allantois forms a singular extension from the hindgut, which exists temporarily between the two umbilical arteries. It regresses from the umbilical cord to connect the hindgut to the cloaca and eventually becomes the ‘urachus’ or ‘umbilical ligament’ (for more information see O'Rahilly and Müller, 1987, Kruepunga et al., 2018, Kalisch-Smith, 2025). In this Review, I use both ‘chorio–allantoic’ and ‘feto–placental’ vasculature terms to describe the equivalent structures between mouse and humans.

Following the onset of oxygenated blood circulation in the embryo after materno–fetal exchange at E10.5 (mouse) and 10 weeks (humans), deoxygenated blood flows from the dorsal aorta to the umbilical artery. As the umbilical artery moves into the placenta, it is continuous with the large placental stem arteries radiating outwards, creating an arterial tree-like structure (Fig. 2, E11.5; Fig. 3, 4+ pcw). These arteries branch further into an expansive capillary plexus for nutrient/gas exchange over the placental barrier from the maternal blood supply (supplied by spiral arteries and returned by venous channels). Fetal capillaries then pass oxygenated blood into large venous vessels, which connect to the umbilical vein, flowing on to the embryonic heart for distribution (Figs 2 and 3). ECs line all the vasculature of the embryo, yolk sac, umbilical and chorio–allantoic placenta, providing vascular structure.

This Review highlights current understanding of feto–placental vascular development, focusing on arterio–venous formation in mice and humans, and illustrates known models exhibiting perturbed formation. I detail major unanswered questions in the placental vascular field, of EC developmental lineage, when arterio–venous formation occurs, and their relevance in human pathologies of pregnancy, including miscarriage, preeclampsia, congenital heart disease and stillbirth. With improvements in genomic technologies, next generation sequencing, as well as organoids and stem cell-based embryo models, solutions to some of these age-old problems are tantalisingly close.

## Development of the placental vasculature

### Composition of the placental barrier in mouse

The mouse has been used as a robust model to test genetic and environmental perturbations of pregnancy. While the mouse placental vasculature is smaller and less branched in structure compared to humans, there are many analogous cell types that perform similar functions. Setting up the materno–fetal interface of the placental barrier, separating maternal and fetal blood supplies, is crucial (Simmons et al., 2008). In mice, this barrier is composed of allantois-derived ECs, two syncytiotrophoblast layers (called SynT-I and SynT-II) and a perforated layer of sinusoidal trophoblast giant cells that line the maternal blood sinusoids of the placenta (Simmons et al., 2008).

### Origin and development of the mouse chorio–allantoic vasculature

The chorio–allantoic blood vessels develop primarily from extra-embryonic mesoderm (Tremblay et al., 2001; Inman and Downs, 2007; Nehme et al., 2025), the differentiation of which occurs over a number of stages before distal attachment to the chorion by E8.5 (Figs 1 and 2) (reviewed by Inman and Downs, 2007; Arora et al., 2012). While incremental steps have been made to understand the formation of the extra-embryonic vasculature, a clear junction between embryonic and allantoic-derived vessels is yet to be defined. For genetic mutations that affect allantois development and differentiation, from allantois budding to chorio–allantoic branching, see Table 1. For gene markers of vascular and vascular-associated cells in mice and humans see Table 2.

**Table 1.** Allantoic and labyrinth mutants due to allantois defects

Gene knockout	Allantois/labyrinth defects	References
**Genes expressed by allantoic mesoderm**		
*T* (brachyury) (chimeras)	Reduced allantoic budding	Rashbass et al., 1991
*Eed*	Overproduction of allantoic mesoderm	Faust et al., 1995; Grosswendt et al., 2020
*T^C^* (T-curtailed)	Reduced budding and growth of allantoic core mesoderm; no allantoic endothelial cell (EC) or plexus formation; aberrant positioning of proximal allantoic vessel (vessel of confluence) connecting umbilical artery/dorsal aorta/vitelline artery; chorio–allantoic defect; embryonic lethal E9.5/E10.5	Inman and Downs, 2006; Rodriguez et al., 2017
*Bmp2*	Short allantois; 50% have chorio–allantoic attachment defect	Ying and Zhao, 2001; Zhang and Bradley 1996
*Bmp4* (KO and chimeras)	Small or no allantois; chorio–allantoic attachment defects	Lawson et al., 1999; Fujiwara et al., 2001; Hadas et al., 2024; Winnier et al., 1995
*Bmp5/7* double KO	Smaller allantois, slightly constricted at base (E8.5); chorio–allantoic attachment defect in 2/3 embryos (E9.5)	Solloway and Robertson, 1999
*Bmp8b*	Reduced allantois length	Ying et al., 2000
*Cdx2*	No/poor chorio–allantoic attachment; chorio–allantoic attachment defect	Chawengsaksophak et al., 2004
*Cdx4* KO compounded with *Cdx2* Het	No/poor chorio–allantoic attachment; no/few invading vessels into the chorion	van Nes et al., 2006
*Eomes*/*T* double KO (chimeras)	No allantoic mesoderm formation	Theeuwes et al., 2024 preprint
*Fak^R454/R454^* (*Ptk2*)	Enlarged allantois; chorio–allantoic attachment defect; haemorrhage; lethal E9.5	Lim et al., 2010
*Foxf1*	Enlarged (hydropic) allantois; chorio–allantoic attachment defect; all of allantois expresses VCAM1 and none with Kdr	Mahlapuu et al., 2001
*Hand1*	Chorio–allantoic attachment defect; lethal by E10.5	Firulli et al., 1998; Riley et al., 1998
*Smad1*	No/poor allantoic budding; chorio–allantoic attachment defect	Lechleider et al., 2001; Tremblay et al., 2001
*Smad5*	Poor allantoic elongation; allantois congregates at proximal pole; chorio–allantoic attachment is unperturbed; majority are lethal by E10.5	Chang et al., 1999
*Isl1*	Reduced allantoic growth/extension; chorio–allantoic attachment defect	Zhu et al., 2024
*Plpp3* (*Lpp3*) (chimeras)	Chorio–allantoic attachment defect; ECs fail to form plexus (allantois explants); enlarged proximal allantois	Escalante-Alcalde et al., 2003
*Mixl1*	Enlarged (hydropic) allantois	Hart et al., 2002
*Tcf1*/*Lef1* double KO	Enlarged allantois; defective placentation by E10.5	Galceran et al., 1999
*Tgfb1*	Chorio–allantoic attachment defect; no *Kdr^+^* progenitors in allantois	Dickson et al., 1995
**Genes expressed by other allantoic progenitors/cells**		
*Fgfr2* (allantois sub-population, not characterised, *Meox2-Cre*)	Chorio–allantoic attachment defect; enlarged (hydropic) allantois; poor invasion of allantoic vessels; lethal by E10/E11	Xu et al., 1998; Molotkov et al., 2017
*Fzd5* (proximal allantois)	Superficial invasion of ECs into chorion; reduced labyrinth vasculature	Ishikawa et al., 2001; Lu et al., 2013
*Hgf* (allantoic mesenchyme)	Reduced labyrinth vasculature; lethal by E16.5	Schmidt et al., 1995; Uehara et al., 1995
*Pdgfrb* (pericytes)	Reduced number of labyrinth pericytes; disorganised labyrinth vasculature; small labyrinth; lethal by E17.5	Looman, et al., 2007
*Mapk14* (*p38a*) (chorionic plate and vessels)	Reduced labyrinth vasculature; lethal by E12.5	Adams et al., 2000
*Tbx4* (allantoic mesenchyme, progenitor to perivascular cells)	Reduced allantoic growth and extension; no umbilical artery or vein formation; chorio–allantoic attachment defect; ECs present but fail to form plexus; chorio–allantoic attachment defect; lethal by E10.5	Naiche et al., 2011; Naiche and Papaioannou, 2003
*Vcam1* (allantois mesothelium, cortex)	∼52-80% allantoises were abnormal; chorio–allantoic attachment defect; enlarged (hydropic) allantois; lethal by E11.5	Gurtner et al., 1995; Kwee et al., 1995
*YAP*/*TAZ* double KO (perivascular cells and ECs, *Hoxa13-Cre*)	Disorganised labyrinth vasculature by E11.5; reduced labyrinth vascular volume at E11.5; reduced peri-vascular cells; lethal by E13.5	Gao et al., 2024 preprint
*Wnt2* (allantois and chorionic plate)	Reduced labyrinth vasculature; hematomas in labyrinth; vascular oedema	Monkley, et al., 1996
**Genes expressed by allantois (not defined)**		
*Hdac1*	Poor allantois formation	Lagger et al., 2002
*Dnmt1*	Enlarged (hydropic) allantois	Li et al., 1992
**Genes expressed by allantois EC progenitors**		
*Etv2* (*Er71*)	Lack ECs, blood vessels and blood formation including allantois; lethal by E9.5	Lee et al., 2008
*Kdr* (*Flk1*)	EC precursors formed but failed to differentiate; lack ECs in the allantois; lethal E8.5/E9.5	Shalaby et al., 1995
*Rspo3* (progenitors and ECs)	Vessels fail to invade placenta; disorganised labyrinth vasculature E10.5; thin labyrinth vasculature; lethal by late gestation	Kazanskaya et al., 2008; Aoki et al., 2007
**Genes expressed by allantois-derived ECs: early lethality**		
*Tgfbr1* (*Alk5*)	Poor invasion of allantoic ECs into chorion; reduced labyrinth vasculature; no smooth muscle around chorionic plate vessels; lethal by E11.5	Larsson et al., 2001
*Apela*	Reduced space of distal attachment of allantois to chorion at E8.5; branching morphogenesis defect; thin labyrinthine vasculature E10.5	Freyer, et al., 2017; Ho, et al., 2017
*Bap1* (*Sox2-Cre*)	Reduced labyrinth vasculature at E9.5; reduced invasion into chorion; reduced branching; lethal ∼E9.5	Perez-Garcia et al., 2018
*Bcas3* (*Rudhira*, *Tie2-Cre*)	Reduced labyrinth vasculature from E10.5; lethal by E10.5	Shetty, et al., 2018
*Cdh5* (*VE-Cad*)	ECs are present but do not form a plexus (allantois explants); no angiogenesis (explants);	Crosby et al., 2005
	Dilated umbilical arteries; lethal by E10.5/E11.5	Gory-Faure et al. 1999
	Allantois-derived ECs did not invade chorionic trophoblast; lethal E9.5/E10.5	Carmeliet et al., 1999
*Dll4* (sprouting ECs and arterial ECs)	Degenerating vasculature in placenta (arterial?); lethality by E10.5	Gale et al., 2004; Duarte et al., 2004
*Mapk3* (*Erk1*) het and *Mapk1* (*Erk2*) KO (*Sox2-Cre*)	Reduced labyrinth vasculature E14.5; lethal by E10.5	Frémin et al., 2015
*Foxo1*	Chorio–allantoic attachment defect; enlarged (hydropic) allantois (E9); lethal by E10.5	Ferdous et al., 2011
*Hey1/Hey2*	Chorio–allantoic attachment defect; poor invasion of allantoic ECs; small placenta at E10.5; lethal from E9.5	Fischer et al., 2004
*Stk11* (*Lkb1*, *Meox2-Cre*)	Reduced labyrinth vasculature E9.5; lethal E9-E11	Londesborough, et al., 2008
*Notch1*/*Notch4* double KO	Allantois-derived ECs didn't invade chorionic trophoblast; lethal by E9.5	Krebs, et al., 2000
*Notch1*	Poor invasion of allantoic ECs into chorion; lethal by E10.5	Limbourg, et al., 2005
*Rbpj* (*Rbpsuh*, *+/− Tie2-Cre*)	Poor invasion of allantoic ECs into chorion; branching morphogenesis defect; lethal from E9.5	Krebs et al., 2004; Lu et al., 2019; Copeland et al., 2011
*Snai1*	ECs fail to form plexus (allantois explant assays); lethal by E10	Lomelí et al., 2009
*Tie2*	Smaller, less branched vessels (allantoic explants); lethal by E10.5	Winderlich et al., 2009; Dumont et al., 1994
***Predicted EC expression***		
Pgap2*, Sqle*	Placental phenotype at E9.5; lethal by E9.5	Perez-Garcia et al., 2018
**Genes expressed by allantois-derived ECs: mid-late-stage lethality**		
*Ctnnb1* (*Tie2-Cre*)	Reduced labyrinth vasculature; smaller umbilical vessels; lethal by E13.5	Cattelino et al., 2003
*Erk2* (*Sox2-Cre*)	Reduced labyrinth vasculature E14.5; lethal by E18.5	Frémin et al., 2015
*Flrt2* (ligand to *Unc5b*, *Tie2-Cre*)	Reduced labyrinth vasculature E12.5; disorganised labyrinth vasculature E12.5; lethal E13.5/14.5	Tai-Nagara et al., 2017
*Ehmt2* (*G9a*, *Tie2-Cre*)	Thin labyrinth; disorganised labyrinth vasculature; increased vascular tortuosity; reduced vasculature from E13.5; lethal by E16.5	Chi et al., 2017
*IP_3_R1* (Itpr1) and *IP_3_R2* (Itpr2) double KO	Reduced labyrinth vasculature at E9.5; lethal by E14.5	Yang et al., 2020
*Map2k1* (*Mek1*) and *Map2k2* (*Mek2*) compound het (*Sox2-Cre*)	Disorganised labyrinth vasculature at E12.5; lethal before birth	Nadeau and Charron, 2014
*Pik3ca* (*PI3K*, *p110α*, *Meox2-Cre*)	Reduced labyrinth vasculature; reduced capillaries; lethal by E12.5	López-Tello et al., 2019
*Rictor* (*Meox2-Cre*)	Reduced labyrinth vasculature by E10.5; developmental delay; lethal by E16.5	Shiota et al., 2006
*Unc5b* (receptor to *Flrt2*, *Tie2-Cre*)	Reduced labyrinth vasculature E12.5; reduced arteriole expansion E12; reversed flow through umbilical artery; lethal by E13.5	Navankasattusas et al., 2008
*Pkd1* (*Meox2-Cre*)	Reduced labyrinth vasculature E14.5; Reduced vascular branching; poor perivascular cell recruitment; perinatal lethal	Garcia-Gonzalez et al., 2010
*Hand1* (*Cdh5-Cre*)	Reduced labyrinth vasculature E16.5; reduced litter size by E18.5	Courtney et al., 2021
***Predicted EC expression***		
Arhgef7*, Cnot4*, Commd10*, Pigf*, Dnajc8*, Nek9*, Nrbp1*, Setd5*	Viable E9.5; placental phenotype detected; lethal E14.5	Perez-Garcia et al., 2018
Chtop*, Fryl*	Labyrinth vasculature defects; placental phenotype detected at E9.5; subviable E14.5; lethal by P14	Perez-Garcia et al., 2018
**Genes expressed by allantois-derived ECs: viable *in utero***		
*Cited2* (*Tie2-Cre*)	Enlarged vasculature E14.5; fetal growth restriction at E18.5	Moreau et al., 2014
*Egfl7*	Reduced area of chorio–allantoic attachment; branching morphogenesis defect; thin labyrinthine vasculature; tortuous capillaries; fetal growth restriction E12.5-E18.5; viable	Lacko et al., 2017
*Igf2* (*Meox2-Cre* and *Tie2-Cre*)	Reduced capillary expansion E16 and E19; fetal growth restriction E19	Sandovici et al., 2022
*Lifr*	No/small labyrinth vasculature; neonatal lethal; 16-20% resorbed after E9.5	Ware et al., 1995
*Pkd2* (*Meox2-Cre*)	Reduced labyrinth vasculature E14.5; reduced vascular branching; poor perivascular cell recruitment; viable	Garcia-Gonzalez et al., 2010
***Predicted EC expression***		
Cir1*, Exoc3l2*, H13*	Labyrinth vasculature defects; viable E14.5; placental phenotype at E14.5; lethal by P14	Perez-Garcia et al., 2018

Asterisk indicates genes predicted to have EC expression in Liang et al., 2021 dataset. The Marsh and Blelloch (2020) dataset was also queried but many genes were only located to the Seurat ‘RNA assay’, indicating very low expression following data normalisation. KO, knockout.

**Table 2.** Mouse and human markers of placental vascular cell types

Cell types	Markers	References
**Mouse labyrinth**		
Allantoic mesoderm	*T* (brachyury), *Fgf8*, *Foxf1*	Nahaboo et al., 2022
Allantoic angioblasts	*Etv2*	Lee et al., 2008
Allantois EC progenitors (∼E8.25)	*Kdr* (*Flk1*), *Sox17*, *Hoxa10*, *Hoxa11*, *Hoxa13*	Ibarra-Soria et al., 2018; Scotti and Kmita, 2012
Hematopoietic stem cells/erythroid progenitors (E9.5+)	CD34, *Runx1*, *Cdh5*, *Sox7*, C-kit, Ter199, CD41	Nahaboo et al., 2022; Ottersbach and Dzierzak, 2005; Zeigler et al., 2006
Hemogenic endothelium	CD44, *Hoxa13*, *Lyve1*, *Runx1*, *Hoxa13*	Liang et al., 2021
Allantoic cortex/mesothelium (E7.5-E8.5)	*Vcam1*	Kwee et al., 1995; Nahaboo et al., 2022
Allantois base (closest to primitive streak)	*Car4*, *Cdh11*, *Stard8*	Nahaboo et al., 2022
Allantois vessel of confluence/allantoic rod	COLIV, PECAM1 (+/−)	Rodriguez and Downs, 2017
Allantoic mesenchyme	*Tbx4*, *Vcam1* (VCAM)	Naiche et al., 2011; Nahaboo et al., 2022
Labyrinth stromal cells	*Col1a1*	Chen et al., 2022
Pan-mural	ACTA2 (αSMA)	Kalisch-Smith et al., 2022
Unknown mural or stromal cells?	PDPN, Gata4, Kit	Marsh and Blelloch, 2020
Pericytes	NG2/CSPG4, ACTA2, *Pdgfrb*, αSMA	Kalisch-Smith et al., 2022; Looman, et al., 2007
Smooth muscle	ACTA2, MYH11 (SM-MHC)	Kalisch-Smith et al., 2022
Pan-endothelial	CD31 (*Pecam1*) CD34, *Cdh5*, *Lyve1* (E12.5-E14.5), *Mest*; laminin, isolectin b4	Kalisch-Smith et al., 2017, 2022
Sprouting ECs	*Dll4*	Nahaboo et al., 2022
Arterial EC	GJA5 (CX40), *Dll4*, *Hey1*, *Hey2*, *Jag1*, EMCN low	Kalisch-Smith et al., 2022; Liang et al., 2021
Artery EC (umbilical)	GJA5	Kalisch-Smith et al., 2022
Arteriole EC	*Dll4*, EMCN low, *Lyve1*?	Kalisch-Smith et al., 2022; Shaut et al. 2008
Venule EC	EMCN high, *Aplnr*	Kalisch-Smith et al., 2022
Vein EC	EMCN high, *Aplnr*, *Nr2f2*	Kalisch-Smith et al., 2022; Liang et al., 2021
Vein	EMCN high	Kalisch-Smith et al., 2022
Capillary EC	*Rtl1*	Sekita et al., 2008
Unknown (EMT?)	PDPN, Isolectin B4^−^	Kalisch-Smith et al., 2022; Marsh and Blelloch, 2020
**Human feto–placenta**		
Extra-embryonic mesoderm	FOXF1, POSTN, VIM, PITX2	Tyser et al., 2021; Zhao et al., 2025; Oldak et al., 2023
Feto–placental EC progenitors	KDR	Aplin et al., 2015
Hematopoietic stem cells/erythroid progenitors	CD34, RUNX1, HOXA9, MLLT3, MECOM, HLF, SPINK2	Aplin et al., 2015; Calvanese et al., 2022
Hematopoietic	CD45, CD235A	Boss et al., 2023
Fibroblast 1	VIM, COL1A2*, DLK1*, CREB3*	Suryawanshi et al., 2018
Fibroblast 2	VIM, COL1A2*, DLK1*, HES1*, TBX2*, REN	Suryawanshi et al., 2018
Fibroblast 3	VIM, COL1A2*, DLK1*, RUNX1*, KLF4*	Suryawanshi et al., 2018
Myofibroblasts	CD26, CD90	Boss et al., 2022
Stromal mesenchyme 1 (abundant <10 weeks)	CD73, CD90, PDPN	Boss et al., 2022
Stromal mesenchyme 2	PDPN, CD36, CD142, CD26, CD90	Boss et al., 2022
Peri-vascular cells	CD146, CD271, CD26, CD90	Boss et al., 2022
Peri-vascular progenitor	CD271	Boss et al., 2023
Pericytes	PDGFRB, αSMA	Harris et al., 2021
Smooth muscle	αSMA	Diehl et al., 2005
Pan-endothelial	PECAM1 (CD31), CD34	Boss et al., 2022
Proliferating ECs	PCNA*, CENPF*, CDC20*	Kalisch-Smith et al., 2024
Arterial EC	GJA5*, HES1*	Kalisch-Smith et al., 2024
Artery (umbilical)	EPHB2	Diehl et al., 2005
Arteriole EC	SOX17*, APLN*, LYVE1*	Kalisch-Smith et al., 2024
Pre-arterial EC/progenitor?	SOX17 low*, KDR*, NRP1*	Kalisch-Smith et al., 2024
Vein/venule EC	APLNR*, NR2F2 (COUPTF-II)	Kalisch-Smith et al., 2024
Vein EC (umbilical)	ADAMTS4, ADAMTS5	Nandadasa et al., 2020
Capillary EC	RGCC*, LYVE1*	Kalisch-Smith et al., 2024
EMT	TAGLN*, ACTA2*, IGFBP7*	Kalisch-Smith et al., 2024

Kalisch-Smith et al., 2024 used human placental single-cell RNA sequencing data from Suryawanshi et al., 2018. Asterisk indicates putative markers (based on clustered gene expression) that have not been confirmed in tissue sections. EC, endothelial cell; EMT, endothelial-to-mesenchymal transition.

#### Budding (∼E7.0-E7.5, late bud stage)

The extra-embryonic mesoderm, which expresses *T* (brachyury) and *Fgf8*, extrudes a bud-like structure (now referred to as allantoic mesoderm) into the exocoelomic cavity towards the chorion (see Glossary, Box 1) (Ellington, 1985; Inman and Downs, 2007; Nahaboo et al., 2022). The allantoic mesoderm extrudes via proliferation and ongoing deposition of mesoderm into the allantois from the primitive streak (Downs and Bertler, 2000; Tam and Beddington, 1987). Mouse genetic knockouts of genes that impact mesodermal proliferation (e.g. *T*, *Bmp4*, etc. see Table 1) prevent allantois budding and/or growth (extension). These knockouts are embryonic lethal due to chorio–allantoic attachment defects, with either a complete absence of or poor placental vascular formation (Lawson et al., 1999; Inman and Downs, 2007). Before allantoic budding, the extra-embryonic mesoderm also gives rise to a thin mesothelial layer that lines the exocoelomic cavity (including the chorionic region), sometimes referred to as ‘chorionic mesothelium’ or ‘chorionic mesenchyme’ (see Glossary, Box 1; Pereira et al. 2011; Nahaboo et al., 2022; Paulo et al., 2011), which has hematopoietic potential (Zeigler et al., 2006).

#### Distal cavitation (3-4 somites)

In order for the allantois to expand and extend toward the chorion, the distal portion of the allantois increases dramatically in extracellular space by cavitation, giving the allantois a foamy-like appearance (Downs and Bertler, 2000). During this time, the allantois accumulates extracellular matrix proteins and proteoglycans including hyaluronic acid. Both groups have protein members that mediate EC tube formation, which may impact on formation of the allantois vascular plexus (see Elongation) (Arora et al., 2012; Crosby et al., 2005). Defects in cavitation have also been associated with aberrant cellular adhesion, another important process for EC tube formation (Naiche and Papaioannou, 2003).

#### Elongation (∼E7.75-E8.25, head-fold stage)

By four-somite pairs, the allantois has elongated and is now covered by a mesothelial layer of squamous epithelial cells (Downs et al.,1998). The mesothelium (*Car4^+^*, *Cdh11^+^* and *Stard8^+^*), also known as the allantois cortex, is located at the distal region of the allantois. The mesothelium begins to express adhesive properties (e.g. VCAM1) that permits future chorio–allantoic attachment (Kwee et al., 1995; Nahaboo et al., 2022). By E8.25, a subpopulation of allantoic mesoderm cells differentiates into *Etv2^+^* angioblasts (Lee et al., 2008), which signal and induce a signalling cascade within *Kdr^+^* (*Flk1^+^*) endothelial progenitors (Downs et al., 1998; Huber et al., 2004) (Table 1). The endothelial progenitors in the allantois form a primitive *Pecam1^+^* endothelial network at ∼E8.25 by vasculogenesis, a process by which new blood vessels are formed (Downs et al., 1998). At this time, in the chorion, the labyrinth trophoblast progenitors (precursors to SynT cells) become patterned and express markers for all trophoblast layers (e.g. *Gcm1*, *Syna*, *Hand1*, *Rhox4b*) (Simmons et al., 2008, reviewed by Simmons, 2013).

#### Chorio–allantoic attachment (E8.25-E8.5, 6-8 somites)

At this stage, the allantoic mesothelium attaches to the chorion (Downs and Gardner, 1995). What happens to the mesothelial layers (covering the allantois and early chorion) after this stage is unknown. Following chorio–allantoic attachment, *Gcm1*^+^ chorionic trophoblast cells (early SynT-II clusters) mark future branch points for the allantois to extend into the chorion (Anson-Cartwright, et al., 2000; Simmons et al., 2008). *Kdr^+^* EC progenitors and definitive ECs are already differentiated before chorio–allantoic attachment, which further expand into the chorion. By E8.5, part of the primary vascular plexus in the allantois also remodels into the umbilical artery (Downs et al., 1998).

#### Invagination (E8.75-E9.0, 9-12 somites)

In order for allantois-derived ECs to be incorporated into the trilaminar labyrinth, these ECs must first invade into the chorion. This process coincides with embryo turning, where the body axis rotates or ‘inverts’ from a concave to convex ‘C’ shaped position. It is hypothesised that cell shape changes and the movement of *Gcm1^+^* chorion cells pass through the chorion, causing it to fold, creating simple branches in which the ECs reside (Simmons et al., 2008). Continued EC branching morphogenesis allows creation of the labyrinth vasculature to support the nutritional needs of the embryos for rapid growth.

#### Branching and maturation (E9.5+)

The umbilical vein is starting to form in the embryo and is connected to the allantois by the 16-somite stage (Walls et al., 2008). How the umbilical vein forms and the progenitors that contribute to its development is currently unknown. Allantoic-derived ECs continue to invade and branch to create the labyrinth vasculature, making a connected arterial and a venous vascular tree (Fig. 2). Many mouse genetic mutations that affect labyrinth EC formation have been investigated and are embryonic lethal at different stages of gestation from E9.5 (Table 1).

### Heterogeneous cell types in the mouse allantois

In addition to the allantois cell types mentioned above, transcriptomic analysis of the allantois has revealed further cell subpopulations at E7.75 (Nahaboo et al., 2022), including *Cdx4^+^* cells in the allantois base (closest to primitive streak), and *Cdh5^+^*/*Sox7^+^*/*Runx1^+^* erythroid progenitors (Nahaboo et al., 2022). A small population of *Dll4^+^* cells have also been found at this age, indicating the presence of early arterial/sprouting ECs (Nahaboo et al., 2022). Compared with other EC populations at E8.25, allantoic EC progenitors express a unique signature of posterior-associated HOXA genes: *Hoxa10*, *Hoxa11* and *Hoxa13* (Ibarra-Soria et al., 2018; Scotti and Kmita, 2012). *Hoxa13* has since been used to engineer mouse Cre/CreERT2 tools to target allantois and placental ECs (discussed below). Further allantois heterogeneity has been shown in a mouse embryonic gastrulation dataset at E8.5, where *Tbx4^+^* allantois cells are distinct from allantoic EC progenitors (Pijuan-Sala et al., 2019). Lineage tracing of *Tbx4:Cre:lacZ* allantoic mesenchymal cells shows that they do not give rise to umbilical ECs but do give rise to support cells surrounding umbilical and placental labyrinth vessels, likely pericytes and smooth muscle (Naiche et al., 2011). Only sporadic placental ECs show labelling at E10.5, although this has not been rigorously quantified.

### Human development of the feto–placental vasculature

In humans, the feto–placental vasculature originates from the connecting stalk and attaches to the chorion from 2 weeks gestation (Figs 1 and 3). Connecting stalk cells extend across the chorionic cavity to make up the feto–placental vasculature and the supporting chorionic plate (see Glossary, Box 1), which contains the major collecting vessels connecting to the umbilical artery and vein (Kalisch-Smith, 2025). In humans, these feto–placental vessels are arranged into a tree-like structure to permit nutrient exchange, and are contained within ‘villi’ – protrusions of trophoblast from the chorionic plate (Fig. 3). The barrier between fetal and maternal blood is composed of two layers of trophoblast cells (syncytiotrophoblast cells in contact with maternal blood and villous cytotrophoblast cells), as well as a layer of ECs. In this way, the human differs from the mouse with the types of cellular layers between maternal and fetal blood supplies (the mouse has two syncytiotrophoblast layers), but the individual cell types (or layers) are functionally similar (Figs 2 and 3). Villous cytotrophoblast cells extend into columns, differentiate and form invasive extravillous trophoblasts, which anchor the villi to the decidua.

The developmental lineage of the connecting stalk and feto–placental vasculature in humans has been unclear, with hypoblast, primitive endoderm, yolk sac and extra-embryonic mesoderm all suggested as potential progenitors. However, recent production and integration of single-cell RNA sequencing (scRNA-seq) of human embryos (Tyser, et al., 2021; Zhao et al., 2025), together with stem cell-based models of post-implantation embryos (Oldak et al., 2023), has revealed new insights. In stem cell-based embryo models created from human embryonic stem cells, cells with an extra-embryonic mesoderm signature (e.g. VIM^+^, POSTN^+^, HAND2^+^, TBX4^+^, HGF^+^), form a connecting stalk towards trophoblast cells across a chorionic cavity at day 8 of culture, equivalent to day 13/14 pcw (Oldak et al., 2023). This extra-embryonic mesoderm signature is similar to that from a human Carnegie stage (CS) 7 embryo (2.5 pcw), expressing FOXF1, POSTN, VIM and PITX2 (Tyser et al., 2021; Zhao et al., 2021 preprint; Oldak et al., 2023) and is the most promising data we currently have in the field as to developmental lineage. While models are yet to show umbilical formation, EC differentiation and chorionic attachment, these will be required in the next iteration of embryo models to ensure developmental competence for later embryonic stages. Further, the genetic signature of extra-embryonic mesoderm in humans mimics non-human primate (cynomolgus macaque) connecting stalk cells (Zhao et al., 2025), in which extra-embryonic mesoderm contributes to placental villous ECs (Jiang et al., 2023).

Endothelial progenitors have been predicted within the connecting stalk from 15-21 days post-conception (from CS5c-6) (Boyd and Hamilton, 1970; Kaufmann et al., 2004; https://www.ehd.org/virtual-human-embryo/), but are yet to be recapitulated in embryo models to determine expression profiles, 3D orientation or exact timing. Culture conditions are likely to influence how these early embryos grow and are yet to fully recapitulate the uterine environment. In the early human placenta, endothelial progenitors have been assessed at 5-9 pcw (CS16-20) and show primitive ‘cords’ located in the chorionic plate and central villus, but not the peripheral villi in which the later feto–placental capillaries develop (Aplin et al., 2015); here, the large stem vessels express markers for EC progenitors (e.g. *KDR^+^*) (Aplin et al., 2015). Vasculogenesis, likely of angioblasts (see Glossary, Box 1), gives rise to these endothelial progenitors of the chorionic plate, while the capillaries arise by angiogenesis of pre-existing blood vessels from 4 pcw (see Kaufmann et al., 2004). Vasculogenesis has also been hypothesised to occur at terminal capillaries from surrounding stromal mesenchyme (Aplin et al., 2015). However, this is yet to be established in human organoid models, which contain stromal mesenchyme but typically lack endothelial cells due to low abundance (compared with trophoblast and stromal cells) and culture techniques that were developed primarily for isolation of trophoblast cells (Turco et al., 2018). Similar experiments could perhaps give insight into ‘vascular pruning’ whereby apoptosis is seen at higher levels in distal capillaries at 7-8 pcw (Tertemiz, et al., 2005). Human organoid and embryo models are likely to yield many insights into early human placental villus formation in the near future.

Even by 4.5 pcw, the human placental vasculature is remarkably complex. Adjacent cytotrophoblast cells produce angiocrine factors, including VEGF, PGF and angiopoetins (Lash et al., 2010), to facilitate vasculogenesis and angiogenesis. When these factors are disrupted, blood vessel formation can be dysregulated and lead to pathological pregnancies (reviewed by Burton et al., 2009). Mesenchymal-derived mural cells are also incorporated into vessels from 4 pcw (Kaufmann et al., 2004). Mesenchymal ‘stromal’ cells, which exist throughout human placental development and maturation (see Glossary, Box 1), consist of mural subtypes including fibroblasts, pericytes and macrophages/Hofbauer cells (Robin et al., 2009; Crisan, et al., 2008), to form the ‘stromal core’ of villi. It is likely that these villous stromal cells are also derived from a common extra-embryonic mesoderm progenitor, before differentiation into a mesenchymal lineage, which is the case in mice and macaques.

## Placental endothelial cell specification

### Specification of mouse allantoic endothelial cell progenitors

As mentioned above, a subpopulation of allantoic mesoderm cells express *Etv2* at E8.25, a marker of angioblasts (Lee et al., 2008). Angioblasts are important in vascular development because they give rise to both endothelial and hematopoietic progenitors and, by doing so, populate many EC populations *de novo* in the embryo (Lee et al., 2008). When *Etv2* is deleted from the embryo, including the allantois, severe blood and vascular defects result (Lee et al., 2008). Specifically, in the chorio–allantoic placenta, *Etv2*-null embryos show an absence of blood vessels and ECs at E9.5 (Lee et al., 2008), indicating that resident allantoic angioblasts are the precursors to umbilical and placental ECs. Furthermore, the expression of *Etv2* and activation of its downstream signalling cascades are important in establishing whether cells differentiate into ECs versus hemogenic lineages. In an experiment creating a range of *Etv2* expression profiles in embryos (combining deletion of an *Etv2* enhancer and crossing it with *Etv2* null or wild-type allele), Sinha and colleagues showed that reducing *Etv2* expression to 20% led to normal EC formation but perturbed erythropoiesis via a reduction in *Tal1* expression (Sinha et al., 2022), a factor with an important role in hematopoietic differentiation (Pijuan-Sala et al., 2019). Using *in silico* analysis, *Tal1* signalling was also predicted to be important in allantois mesoderm allocation (Kamimoto et al., 2023). Here, authors designed a genome regulatory network programme called CellOracle, which allows *in silico* perturbation of cell lineage-specifying transcription factors. Kamimoto and colleagues predicted that *Tal1* knockout increases allantoic mesoderm at the expense of hemato–endothelial progenitors (Kamimoto et al., 2023). Overproduction of allantoic mesoderm is also seen in another mutant for *Eed*, a component of the PRC2 complex (Faust et al., 1995; Grosswendt, et al., 2020). Altogether, this suggests that there is fine genetic control of cell allocation to the allantoic mesoderm and hematopoietic lineages. Use of CellOracle (Kamimoto et al., 2023) to predict genome regulatory networks and allantois phenotypes could yield further interesting insights into important transcription factors at play during chorio–allantoic formation.

### Human endothelial cell specification

Profiling of human endothelial cells in scRNA-seq datasets shows that the embryonic dorsal aorta is already differentiated by CS13 (4 pcw) (Xu et al., 2023), while the fetal heart has arterial and venous populations by 13/14 pcw (McCracken et al., 2022). Placenta arterio–venous differentiation is likely to be matched with that occurring in the embryo and is, therefore, likely to occur much earlier than currently thought. Although not necessarily a surrogate of placental EC formation, the umbilical cord starts to form arterial vessels before CS9 and venous vessels from CS11 (∼3.5 pcw) (Kalisch-Smith, 2025), which is earlier than previously specified (O'Rahilly and Müller, 1987). In light of the new computational lineage trajectories discussed above, and umbilical differentiation being earlier than anticipated, it would appear more likely that placental and umbilical ECs are developing concurrently from connecting stalk EC progenitors, rather than developing independently before connecting to each other, as previously thought. Additional scRNA-seq datasets available from the first trimester human placenta (Suryawanshi et al., 2018; Vento-Tormo et al., 2018) will hopefully shed more light on early placental vascularisation in combination with synthetic embryo models.

## Two-way communication between the labyrinth trophoblast and endothelial cells

Key to the continued formation of the placental vasculature in both mice and humans, is communication of ECs with the adjacent chorionic trophoblast. Although in other vascular beds, secreted pro-angiogenic signalling (e.g. VEGFA) comes from cardiomyocytes (heart), chondrocytes (bone), podocytes (kidney), neural tube and macrophages (Coultas et al., 2005), in the placenta, it is the chorionic trophoblast that provides these signals. In the mouse, the labyrinth trophoblasts progenitors and later SynT-II cells produce VEGF to initiate sprouting angiogenesis (EC production from existing blood vessels) via its receptor KDR (VEGFR2; Nahaboo et al., 2022; Marsh and Blelloch, 2020; Simons et al., 2016). Trophoblast paracrine signalling also has an important additional role: to match the development of the labyrinth vasculature with growth and remodelling of the maternal side of the placenta. Experiments in mice show that poor expansion of the spiral arteries and maternal blood spaces (sinusoids) can lead to reduced expansion of the fetal capillaries and impact the transport of nutrients to the fetus, ultimately causing fetal growth restriction (FGR) (Hamada et al., 2007; Kalisch-Smith et al., 2019). Furthermore, in humans it is well established that aberrant angiocrine signals from trophoblast cells can also lead to preeclampsia, a condition defined by poor spiral artery remodelling, hypertension at >20 weeks gestation and maternal endothelial dysfunction (reviewed by Dimitriadis et al., 2023), which also impacts the feto–placental vasculature (discussed below).

In mice, labyrinth trophoblast progenitors also contain corresponding ligands or receptors to those derived from allantoic ECs. For example, the allantois expresses VCAM1, whereas the chorion expresses its receptor integrin α4, thus permitting chorio–allantoic attachment (Kwee et al., 1995). Similarly, other receptor–ligand interactions are involved, including APELA (ELABELA)–APLNR required for angiogenesis (Freyer, et al., 2017; Ho, et al., 2017) and WNT–FZD (Nahaboo, et al., 2022; Parr et al., 2001). WNT signalling is required for SynT-II differentiation (Zhu et al., 2017), with allantois-derived *Wnt2* deletion reducing labyrinth formation (Monkley, et al., 1996), and deletion of chorion-derived *Wnt7b* showing poor chorio–allantoic attachment (Parr et al., 2001). WNT–FZD5 signalling is also important for upregulation of *Vegf* expression in chorion trophoblast (Lu et al., 2013), forming a positive feedback loop. Recent discoveries from scRNA-seq of E9.5-E14.5 placental labyrinths has revealed known and previously unreported interactors between the chorion/labyrinth trophoblast/SynT-II with allantois ECs (Marsh and Blelloch, 2020). These include known interactors, such as VEGFA–KDR and VEGFA–FLT1, and previously unreported interactions to play a role in placental vascularisation, such as LGR5–RSPO3. RSPO3 is part of the WNT/β-catenin signalling pathway and its deletion is embryonic lethal due to defective chorio–allantoic attachment (Aoki et al., 2007). With the generation of these new scRNA-seq datasets from placental labyrinth development (Marsh and Blelloch, 2020; Liang et al., 2021), undoubtedly, more receptor–ligand interactions will be uncovered, which can be further tested in genetic mouse models. A challenge in the placental field in determining new interactions will be the reliance on available receptor–ligand libraries, which are curated based on known interactions in embryonic and adult contexts. Given the unique cell types within the placenta, creation of placental proteomic and protein–protein interaction datasets would be a good start to curate placental-specific libraries.

## Vascular defects

### Chorio–allantoic attachment, a necessary checkpoint in mouse development

A major checkpoint in placental development is at the chorio–allantoic attachment phase: if not achieved this leads to embryonic lethality (Copp, 1995). Indeed, many mouse mutants exhibit poor/absent allantoic vascular formation, including defects in attachment upon perturbation of *Tbx4* (Naiche and Papaioannou, 2003), *Fgfr2* (Xu et al., 1998) and *Mtrr* (Wilkinson et al., 2021), as well as errors in initial sprouting of ECs into the chorion from mice null for *Grb2* (Saxton et al., 2001), *Fzd5* (Ishikawa et al., 2001; Lu et al., 2013), *Notch1* (Krebs et al., 2000; Limbourg, et al., 2005), *Hey1*/*Hey2* (Fischer et al., 2004) and *Rbpj* (Lu et al., 2019). Intriguingly, the *Mtrr* hypomorph also showed eccentrically located placentas in 12% of embryos, where allantoic and umbilical insertion was not in the centre of the chorion (Wilkinson, et al., 2021). The allantois can also vary in the space it uses to attach to the chorion, with the knockout of *Apela* showing reduced distal attachment at E8.5 (Freyer, et al., 2017). A different study showed that knockout of the same gene caused poor feto–placental angiogenesis and a thin labyrinthine layer later at E10.5 (Ho, et al., 2017), demonstrating that defects during early stages can have long-lasting impacts.

Continued labyrinth development and vascular branching requires further communication between the chorion and allantoic-derived ECs. Chorion-expressed *Gcm1* cell clusters mark future branch points for the allantois to extend into the chorion (Anson-Cartwright, et al., 2000; Simmons et al., 2008). *Gcm1* expression appears before chorio–allantoic attachment but requires allantoic contact for expression to be maintained; for example, through WNT–FZD-GCM1 signalling (Hunter, et al., 1999; Lu et al., 2013). Further labyrinthine branching also requires activation of cMet (expressed on labyrinth trophoblast progenitors) by allantoic mesenchyme-derived HGF (Ueno et al., 2013; Uehara, et al., 1995). In a large mouse knockout screen of 103 mutants, ∼40% were lethal between E9.5 and E14.5, and 95% of these had severe placental phenotypes at E9.5 (Perez-Garcia, et al., 2018). These observations demonstrate how crucial the early stage of placental development is for embryo–placental competence in later gestation, and that without sufficient formation of these allantoic-derived vessels, this likely results in embryonic lethality. For further allantois vascular mutants impacting feto–placental vascular formation, see Table 1 (Arora et al., 2012; Woods et al., 2018).

### Placental mural cells, another cause of embryonic lethality

In mice, embryonic lethality also results from poor pericyte and smooth muscle recruitment from allantois-derived cells. *Pdgfb^+^*/*ASMA* (*ACTA2*; alpha smooth muscle actin)^+^ pericytes surround ECs and provide necessary structural and metabolic support via the release of growth factors. When *Pdgfb* or *Pdgfrb* is deleted, pericyte differentiation is impaired and the labyrinth vascular organisation is disrupted from E13.5 (Ohlsson et al., 1999; Looman et al., 2007). ASMA*^+^*/MYH11*^+^* (SM-MHC) smooth muscle cells surround arterial and arteriole ECs of the placental labyrinth and other chorionic plate cells by E15.5 (Navankasattusas et al., 2008; Kalisch-Smith et al., 2022). Smooth muscle cells are yet to be extensively investigated for cell autonomous impacts in genetic mouse models. When these mural cells arise, and from which progenitors, is yet to be investigated.

### Do early feto–placental vascular defects underlie miscarriages in the first trimester?

One pertinent question to be answered in humans is whether chorio–allantoic defects occur in humans as they occur in mice, and whether these are a cause of early miscarriages. In mice, these defects are a common cause of lethality and a checkpoint for successful development. Even poor initiation of vascular branching early in mouse gestation has far-reaching consequences for the remainder of development and embryo competence. A recent study of miscarriages occurring at 7-10 pcw showed delayed embryo development, with embryos being four Carnegie stages earlier than those in an ongoing pregnancy, as estimated by crown–rump length (Pietersma et al., 2023). Whether the human placenta is equally affected during this early stage, and perhaps the cause of these miscarriages, however, is unknown. With new embryo models available, investigation of chorio–allantoic attachment and assessment of gene knockouts in human development feels closer than ever and would be inconceivable without this technological advance.

Investigation of placental defects during the first trimester from implantation are often assumed to be due solely to poor trophoblast formation and function, with little or no insight into placental vascular formation. To begin to explore this topic, we have recently carried out a systematic review of endothelial genes (gene-associated single nucleotide polymorphisms) from genome-wide association studies, involved in pregnancy disorders including miscarriage, stillbirth and congenital heart defects (CHD) (Kalisch-Smith et al., 2024). As cardiac and placental ECs share many of the same gene programmes, we assessed their likelihood of either heart or placental ECs being the cause of these pathologies. We first found that ∼7% of EC-associated genes were associated with miscarriage, 4% for CHD and 3% for stillbirth (Kalisch-Smith et al., 2024). Roughly half were more likely to be attributed to the placental endothelial expression (versus heart) for both miscarriage and CHD, compared with only 30% for stillbirth, with 70% being more likely to be attributed to heart-expression of EC genes (Kalisch-Smith et al., 2024). Further investigation into placental EC genes is now of utmost importance to understand their expression profiles, and their contribution to placental development and disease.

## Open questions in placental arterio–venous formation

### Placental endothelial specification – mouse versus human

Although it is clear that allantoic tissue differentiates into the *Efnb2^+^* umbilical artery by E8.5 and the *Ephb4^+^* umbilical vein by E9.5 (Wang et al., 1998) in mice, it is unclear how – and when – the feto–placental ECs are formed. Blood flow can be seen by E10.5 in the umbilical cord, which suggests that the arterio–venous circulation is already connected and functional. Placental arterio–venous differentiation is complete by E12.5, shown by the presence of mature markers of placental arteries [e.g. *Gja5* (*Cx40*) and *Dll4*] and veins (e.g. EMCN and APLNR) (Kalisch-Smith et al., 2022). In addition, *Vegfc* and *Aplnr* (*Apj*) are expressed in a subset of vessels at E9.5/E10.5 (Outhwaite et al., 2019; Freyer et al., 2017). Despite the robust expression of many arterio–venous genes in placental ECs, their roles in the placental vasculature are rarely investigated. To date, only two studies have investigated a placental phenotype when knocking out an arterial gene (*Dll4*; Gale et al., 2004, Duarte et al., 2004), while no studies deleting venous-associated genes have investigated the placenta.

*Dll4* is a ligand to NOTCH receptors and is expressed in both embryonic arterial and sprouting ECs (Chong, et al., 2011). Haploinsufficiency of *Dll4* shows degenerating placental vessels, particularly in the large stem arteries, and embryonic lethality by E10.5 (Gale et al., 2004; Duarte et al., 2004). Considering that chorio–allantoic attachment also occurs from E8.5 (Fig. 1), we could predict that the placental arteries bud from the umbilical artery, while the placental veins bud from the umbilical vein. However, contradictory to this theory, in the embryo, arterial ECs are not proliferative and are formed by alternate mechanisms. Although proliferation is not seen in major embryonic arterial ECs (with high expression of *Unc5b*, *Dll4* and *Efnb2*), higher proliferation is seen in arterioles and venous capillaries (Hou et al., 2022). In models of injured adult arteries, arterial ECs must de-differentiate before proliferation and re-assembly into arteries (Das et al., 2019). An alternative way to generate arterial ECs, used by the sinus venosus (venous progenitor in the heart), is through the proliferation of venous ECs, which then differentiate into the coronary arteries (Red-horse et al., 2010; Su et al., 2018). Whether specific intermediates are required for placental EC development from the umbilical cord vessels is now of great interest. Furthermore, the cellular origin and exact timing of umbilical vein development is yet to be fully established.

### The placental vasculature – is it a transcriptionally unique endothelial bed?

A major disadvantage in the placental field is that scRNA-seq datasets are created on placental tissue in isolation, without incorporation with other embryonic/extra-embryonic tissues. Without these, it is very difficult to integrate datasets together without overlapping cell populations. To understand whether the placental vasculature is unique, for example compared with embryonic or yolk sac vascular beds, additional datasets are required. Currently, the transcriptional pathways used to create the placental vasculature, both *de novo* from mesoderm (i.e. vasculogenesis) as well as angiogenic growth of existing blood vessels, are similar to systems in both the embryo and yolk sac. These conserved factors and pathways for formation of mesoderm (e.g. *T* and *Bmp4*), differentiation to EC progenitors (e.g. *Etv2* and *Kdr*) and angiogenesis (e.g. *Tie2*, *Cdh5*, *Vegf*, etc.) show that the placenta is equally affected by these processes (Table 1). That said, subtle differences between ECs of the placenta versus other organs are starting to emerge. For example, a study in human tissue from around 10-18 pcw has shown placental ECs do have a unique transcriptional profile compared with other organ vascular beds (Cao et al. 2020). Here, placental ECs express *LHX6* (encoding for a unique cysteine-rich zinc-binding domain), *LIN28B* (encoding a protein that regulates mRNA translation and miRNA maturation) and *MEOX2* (a transcription factor). These genes are yet to be investigated in detail and may provide the placenta with additional adaptability in adverse environments to control vessel formation or function. Subtle changes between embryonic and placental NRP1 and NRP2 expression have also been found (Kalisch-Smith et al., 2022). While embryonic NRP1 is expressed in arterial ECs (Chong et al., 2011; Erskine et al., 2017), placental NRP1 is expressed in arterial ECs at E12.5 but not E15.5 (Kalisch-Smith et al., 2022). Similarly, while embryonic NRP2 is expressed in venous ECs (Chong et al., 2011), in the placenta NRP2 is only expressed in a small subset of ECs (Kalisch-Smith et al., 2022). Additional transcriptional differences are likely, given that the placental arterio–venous system carries blood with different oxygenation to the embryo (e.g. placental arteries have deoxygenated blood, embryonic arteries have oxygenated blood). Furthermore, given that placental ECs are directly adjacent to trophoblast cells, ECs could adopt a different profile to signal with these trophoblast cells, either by direct contact or a paracrine manner. While mouse studies exploring placental endothelial heterogeneity versus other embryonic endothelial beds are yet to be performed or integrated with available datasets, an organotypic transcriptional signature is likely given the above outcomes and the high degrees of endothelial heterogeneity found in early mouse embryos (Hou et al., 2022) and from other adult organs (Wang et al., 2022).

### Placental arterio–venous vascular malformations in disease

#### Mouse models

It is well established in mice that both placental arterial and venous networks can change in response to genetic and environmental perturbations. The arterial tree is reduced in size by knockout of *Unc5b* (binds *Flrt2* described later; Navankasattusas et al., 2008) or is enlarged by hypomorphic *Gcm1* (Bainbridge et al., 2012), exposure to cigarette smoke (polycyclic aromatic hydrocarbons) (Detmar et al., 2008) and lower diversity of the maternal microbiome (Pronovost et al., 2023). Conversely, knockout of *Cited2* reduces the expansion of both arterial and venous trees (Withington et al., 2006). The arterial circulation is also impacted in mouse embryos with CHD caused, by example, from maternal iron deficiency (Kalisch-Smith et al., 2021, 2022). If arteriole development is constricted, for example by reduced blood flow from the heart, blood flow may be reversed and cause embryonic lethality (Navankasattusas et al., 2008; Tai-Nagara et al., 2017*).* Although arterio–venous defects have not been well studied, in general, the feto–placental vasculature is reduced or disorganised in many mouse genetic knockout models that cause CHD, such as *Ncx1* (*Slc8a1*; Cho et al., 2003), *Cxadr* (Outhwaite et al., 2019), *Flrt2* (ligand for *Unc5b* mentioned above; Tai-Nagara et al., 2017) and excess glucocorticoids (made by knocking out *Hsd11b2* expression, which inactivates glucocorticoids; Wyrwoll et al., 2016), among others (Maslen, 2018; Camm et al., 2018). In the cases of iron deficiency and *Cxadr* loss-of-function embryos (in which *Cxadr* has been deleted using *Sox2-*Cre, which targets epiblast derivatives, including allantoic blood vessels), the heart defects preceded the placental vascular defects. Conversely, in the *Flrt2* mutant, placental vascular defects preceded the heart defects. Further, a small placental vascular bed mimicked by occlusion of the outflow tract (using experiments conducted in the chick) can cause a range of CHD defects depending on the level of constriction (Midgett et al., 2017). Curiously, mouse genetic knockouts that primarily impact placental SynT-I cells can also cause CHD (Perez-Garcia, et al., 2018; Radford et al., 2023). CHD could, therefore, be due to primary or secondary heart defects, because heart defects cause placental defects (Outhwaite et al., 2019; Kalisch-Smith et al., 2021, 2022; Radford et al., 2023), and placental defects cause heart defects (Barak et al. 1999; Adams et al., 2000; Perez-Garcia, et al., 2018; Ward et al., 2023; Radford et al., 2023; Fan et al., 2024).

It has not yet been well explored how mutations that affect SynT-I or SynT-II can elicit heart defects. A few possibilities could include knock-on impacts to the differentiation and development of adjacent SynT-II with the EC layer, which would contribute to heart defects through reduced flow. Another possibility, could be secreted trophoblast factors that make their way into the fetal circulation, impacting heart and embryonic development. An interesting avenue would be to examine how mutations that affect SynT-I/SynT-II impact the formation of the arterial and venous vascular trees. Given that trophoblasts are adjacent to both these vascular trees, it has not been considered whether the trophoblast is patterned differently, next to venous/arterial/capillary ECs, and how these interactions could reciprocally impact trophoblast development. Spatial transcriptomics experiments may offer some insight. In addition, given that labyrinth progenitors and SynT-II cells secrete VEGFA, a signal that confers both angiogenesis and arteriogenesis (at high levels) (Pontes-Quero et al., 2019; Luo et al., 2021), how does this spatially pattern and expand the arterial tree, capillary plexus versus the venous tree? It would also be interesting to understand what degree of placental vascular constriction confers CHD and other heart phenotypes such as coronary vessel development, myocardial proliferation etc. This may help us understand why embryos die at specific gestational ages. For example, coronary arteries recruit smooth muscle from E14.5, which, if affected, causes coronary arterial defects and embryonic lethality (reviewed by Smart et al., 2009; Lupu et al., 2020).

#### Human evidence of vascular defects

Similar to the mouse, human pregnancies with CHD often show placental defects, such as delayed villous maturation and poor perfusion (Matthiesen et al., 2016; Jones et al., 2015; Rychik et al., 2018; Courtney et al., 2020; O'Hare et al., 2023; Mahadevan et al., 2023). Women with preeclamptic pregnancies before 34 weeks of gestation, also have a 7-fold increased risk of their baby developing CHD (Boyd et al., 2017). Placental tissue from preeclamptic pregnancies exhibit feto–placental vascular defects including avascular villi, fetal vascular malperfusion and narrower umbilical cords, among other villous defects including villous hypoplasia, infarction, syncytial knots and perivillous fibrin deposition (Leavey et al., 2016; Benton et al., 2018). Villous hypoplasia is suspected to lead to poor vascular development and ultimately results in reduced placental weight and FGR (Fitzgerald, et al., 2012). These vascular defects are thought to be secondary to trophoblast defects, characterised by aberrant S-FLT and PGF levels – two important angiogenic proteins. Biphasic effects to both trophoblast and endothelium are not surprising considering the important angiocrine signals the trophoblast secretes (along with VEGF) to facilitate feto–placental vascular development as well as vascular remodelling on the maternal side of the placenta (Zhou et al., 2002). Feto–placental vascular defects including poor villous maturation, perfusion and reduced villous stromal cells are also common in pregnancies with FGR (Tun et al., 2019*;* Boss et al., 2023) and stillbirth (Wu et al., 2021; Tiwari et al., 2021; Stallmach et al., 2001). Indeed, 50% of stillbirths are also preceded by intrauterine growth restriction (IUGR) (Figueras and Gardosi, 2011), with 20-60% of stillbirths associated with placental abnormalities (Stillbirth Collaborative Research Network Writing Group, 2011; Varli et al., 2008; Korteweg et al., 2009). Feto–placental blood vessels can also be affected by maternal environmental impacts including iron-deficiency anaemia, gestational diabetes mellitus, smoking, IUGR and FGR (Mayhew et al., 2004).

#### Diagnostic imaging of feto–placental vascular defects

Studies of vascular formation *in vivo* in humans are limited by technology and resolution of imaging modalities. Doppler ultrasound can give an overall measure of placental size and utero–placental blood flow in the first trimester scan (Mathewlynn and Collins, 2019), but is yet to estimate regions of the feto–placental tree. That said, first trimester placental volume has become an accurate predictor of conditions including preeclampsia with gestational hypertension (Hashish et al., 2014) and FGR (Papastefanou et al., 2018; reviewed by Srinivasan et al., 2021). Analysis of the feto–placental tree *in vivo* during early gestation, however, appears to be many years away.

Investigation into human placental arterio–venous vascular defects is currently limited. Current efforts on term placentas use vascular casts (Junaid, et al., 2017) or microCT (Byrne et al., 2021; James et al., 2021). Placentas from pregnancies with FGR show reduced arterial vessels but increased venous vessels (Junaid, et al., 2017). Another study showed that IUGR placentas had reduced arterial and venous branch radii (Saw et al., 2018). The causes of such defects could include poor proliferation or perturbed initial specification of cells to either population, or limited expansion of the arterial or venous trees from reduced/perturbed blood flow. Indeed, deletion of arterial *Dll4* in mice reverts ECs to a venous identity (Duarte et al., 2004), which would perhaps explain an increase in venous vessels at the expense of arterial ones. Similarly, in mice, the placental arterioles appear to be more sensitive to blood flow changes i.e. as a result of heart defects (as previously mentioned in mouse models) and are more affected than the venous circulation in the placenta. The umbilical vessels may give clues to placental defects, as they connect to the respective placental arterial and venous vasculature, and abnormal cord insertion is associated with FGR, placental abruption and fetal demise (Moshiri et al., 2014). Further, 10% of stillbirths are associated with umbilical cord abnormalities (Stillbirth Collaborative Research Network Writing Group, 2011). In the context of IUGR, examination of the umbilical vein shows reduced size and structural alterations (Rigano et al., 2008; Peyter et al., 2014). Alternatively, the umbilical artery shows reduced flow in babies before delivery when the terminal villi are maldeveloped (Krebs et al., 1996). For a complete review of umbilical circulation and remodelling in mice and humans see Downs (2022) and Van Schoor et al. (2024), and for pathophysiology see Burton and Jauniaux (2018).

## Tools and techniques for studying feto–placental blood vessels: problems in genetic targeting of mouse placental ECs

Conventionally, *Sox2-Cre* and *Meox2-Cre* have been used to target cells from epiblast derivatives, such as embryonic lineages and extra-embryonic mesodermal lineages including the allantois (but not, for example. yolk sac visceral endoderm), which makes it difficult to determine the causality in genetic knockout studies. Embryonic defects impacting the systemic vasculature and causing lethality could therefore be attributed to several organs and cell types including the heart, yolk sac and placenta. *Sox2*-Cre and *Meox2*-Cre approaches have been used in combination with trophoblast-lineage Cres (*Cyp19a1* for pan-trophoblast, *Tpbpa* for junctional zone lineages or *Sox2*-FLP for pan-trophoblast lineages) to compare phenotypes from genetic deletions from epiblast-derived lineages versus trophoblast lineages (López-Tello et al., 2019; 2023; Radford et al., 2023).

Until recently, we have lacked genetic tools to research allantois lineages (or placental ECs) specifically in mice. However, as previously mentioned, recent studies have determined the constitutive *Hoxa13-Cre* allele to be allantois-specific, expressed highly in allantoic progenitors (Chen et al., 2022) along with other HOX genes (i.e. *Hoxa10* and *Hoxa11*) at E8.25 (Ibarra-Soria et al., 2018; Scotti and Kmita, 2012). This has, therefore, been a very effective Cre allele for lineage tracing of allantois derivatives; ECs and mural cells (Chen et al., 2022). To target this *Hoxa13* lineage during specific developmental windows of placental formation, a tamoxifen-inducible Cre was created (*Hoxa13*-CreERT2). However, using this Cre to target all placental vessels is not appropriate because *Hoxa13* becomes restricted in expression to the umbilical artery and a subpopulation of the major placental arteries following differentiation of allantoic EC progenitors, and it is also expressed in limb buds (Liang, et al., 2021). Therefore, both of these systems may not be good models with which to generate placental EC-specific conditional knockouts. If umbilical ECs are the source of placental ECs, a constitutive Cre approach that targets all allantois derivatives will presumably perturb initial placental EC formation and cause chorio–allantoic attachment defects and embryonic lethality in every model by E9.0-E9.5, preventing later analysis. Conversely, an inducible knockout approach targeting only umbilical and placental stem arteries leaves capillary and venous EC understanding lacking.

In addition, *Isl1*-Cre has also been used to target allantois derivatives because it is expressed in extra-embryonic mesoderm (Zhu et al., 2024); however, *Isl1* is also expressed in embryonic pharyngeal mesoderm and the second heart field of the developing heart (Cai et al., 2003), so cannot be used in a placental EC-specific manner. Therefore, additional genetic tools are now required to complement existing models to better progress our understanding of these processes. Additional genetic tools, such as intersectional genetics, may overcome the problems above and provide ‘placental-EC-specific’ gene deletion. For example, if a generic allantois-Cre, such as *Hoxa13*-Cre (Cre-lox), is crossed with an endothelial Dre (Dre-rox) line, such as *Cdh5-Dre* (Han et al., 2021) or *Tie2-Dre* (Pu et al., 2018), this will provide deletion by excluding cells not co-expressing Cre and Dre. Together these tools may provide us with more information on how placental defects can cause heart defects, amongst other embryonic vascular, and perhaps even lymphatic, outcomes if a systemic approach is pursued.

## Conclusions

The feto–placental blood vessels originate from extra-embryonic mesoderm in mice and this is likely to be the case in humans. Arterio–venous differentiation occurs by mid-gestation, with early specification likely taking place around CS11-13 in humans, and by E10.5 in mice. New scRNA-seq datasets are likely to shed light on arterio–venous specification and differentiation and confirm the progenitor populations from which they derive. Defects in early placental EC formation cause embryonic lethality in mice; however, this is yet to be established in humans. Poor development of the feto–placental blood vessels and arterio–venous vasculature could have early origins in the first trimester and should be the focus of future studies.
